# Advances in CT Techniques in Vascular Calcification

**DOI:** 10.3389/fcvm.2021.716822

**Published:** 2021-09-29

**Authors:** Lijie Zhang, Lihua Li, Guoquan Feng, Tingpan Fan, Han Jiang, Zhongqun Wang

**Affiliations:** ^1^Department of Cardiology, Affiliated Hospital of Jiangsu University, Zhenjiang, China; ^2^Department of Pathology, Affiliated Hospital of Jiangsu University, Zhenjiang, China; ^3^Department of Radiology, Affiliated Hospital of Jiangsu University, Zhenjiang, China

**Keywords:** vascular calcification, calcification score, micro CT, dual energy CT, multi-slice spiral CT

## Abstract

Vascular calcification, a common pathological phenomenon in atherosclerosis, diabetes, hypertension, and other diseases, increases the incidence and mortality of cardiovascular diseases. Therefore, the prevention and detection of vascular calcification play an important role. At present, various techniques have been applied to the analysis of vascular calcification, but clinical examination mainly depends on non-invasive and invasive imaging methods to detect and quantify. Computed tomography (CT), as a commonly used clinical examination method, can analyze vascular calcification. In recent years, with the development of technology, in addition to traditional CT, some emerging types of CT, such as dual-energy CT and micro CT, have emerged for vascular imaging and providing anatomical information for calcification. This review focuses on the latest application of various CT techniques in vascular calcification.

## Introduction

In hypertension, diabetes, atherosclerosis, and other diseases, vascular calcification, which severely affects human health, must not be ignored (1). Currently, there are many quantitative methods to calculate a vascular calcification level score. The most commonly used process is the Agatston (2), in which lesions with CT value ≥130 Hu and area ≥1 mm^2^ (3) are defined as calcification. It is worth noting that the Agatston score is for the assessment of calcification in the coronary artery. As a clinically accessible imaging technique, CT is the most advanced non-invasive tool for detecting coronary calcification (4). CT can quickly and easily image and analyze the calcification. Bradley et al. (5) divided calcification into three characteristics: perimeter, length, and morphology, and the score accorded each feature. Multiple studies (6) have verified the accuracy and importance of calcification score derived by CT. In this review, recent development in various CT techniques in evaluating vascular calcification is stated. Moreover, we discuss the application and future development of CT in vascular calcification.

## The Significance of Vascular Calcification Assessed by CT, Especially in the Coronary Artery

CT is a non-invasive imaging method for evaluating and analyzing vascular calcification. It is considered the gold standard for analyzing and quantifying vascular calcification (7, 8). Many studies have shown that the coronary calcium score is an essential and reliable predictor as levels of calcium increase with the morbidity and mortality of cardiovascular disease in patients (9–11). In a feasibility study, patients with a calcium score over 400 had a high risk of cardiovascular disease, while those who scored 1 to 400 were at twice the risk of cardiovascular disease as those with a zero score (12). Rennenberg et al. analyzed the results of 30 different studies. They found a three to four times higher risk of incidence and mortality of cardiovascular disease in patients with vascular calcification (13).

Coronary calcium scoring is useful for a wide range of factors such as age and risk. It can assist risk stratification for clinical cardiovascular events (14, 15). Furthermore, the coronary artery calcium score (CACS) helps improve risk prediction in intermediate-risk groups when combined with other traditional clinical risk classification methods (16). This was confirmed by Tamar et al. (17). They found a greater redistribution of risk for middle-grade patients. This suggests that middle-risk patients may be more appropriate for coronary calcium scores. CACS was verified as optimizing the risk classification of cardiovascular events. It has significantly improved the accuracy of cardiovascular risk stratification. More importantly, CACS can help some patients at risk of cardiovascular disease be treated with statins at a secondary prevention level (18).

As a relatively inexpensive non-invasive imaging method (19), coronary computed tomography (CCTA) has become a vital examination method of patients with coronary disease in the clinical diagnosis and treatment plans due to its advantages of high spatial resolution, high sensitivity (95–99%), and high negative predictive value (97–99%) (20). The resolution of currently available CCTA is 0.5–1 mm, and CCTA is widely used to prevent and examine coronary heart disease (21). As a non-invasive examination method, compared with invasive coronary angiography, the visualization of blood vessels is the main advantage of CCTA (22). It provides information about coronary arteries' structure and information about the shape and composition of vascular plaques. Furthermore, the severity of coronary plaques estimated by imaging is consistent with intravascular ultrasound (IVUS) (23). However, it cannot detect minute elements such as macrophage accumulation and prominent plaque characteristics (24).

Using the traditional CT scanner, coronary artery calcium (CAC) is defined as (3) a lesion above the threshold of 130 sound field units with an area ≥1 mm^2^. Studies have shown that (25) for lesions with calcification score ≥400, the sensitivity and specificity of conventional CT in the diagnosis of coronary heart disease are lower than those of CCTA technology. Furthermore, as CCTA provides less information when the calcification score is high, severely calcified plaques cause beam hardening and blooming artifacts, resulting in inaccurate diagnoses of coronary artery stenosis (26). Skinner et al. (27) recommended invasive coronary angiography for patients with a CAC score >400. It has been proved that (28) adding the transluminal attenuation gradient of a transverse optical lumen to CCTA can improve the diagnostic accuracy of CCTA. Experiments show that (29) a higher heart rate will affect the repeatability of CCTA plaque measurement. Higher heart rates will produce motion artifacts, leading to poor plaque image quality. Some strategies have been developed to improve CCTA diagnostic performance in calcified plaque recognition (30, 31). These methods include using image post-processing methods and iterative reconstruction (IR) algorithms to suppress the influence of severe calcification on coronary artery lumen evaluation. Li et al. (32) found that the blooming removal algorithm significantly reduced blooming artifacts caused by calcified plaque, reduced the occurrence of false-positive coronary artery lesions, and improved CCTA diagnostic accuracy. It is worth noting that a study found that (33) the relationship between calcium volume and density and subsequent clinical diseases varies. Coronary artery calcification volume is positively correlated with both coronary heart disease and cardiovascular disease, while calcification density is inversely proportional to both. Several other studies have supported this view, concluding that stabilization has a greater density of calcification plaques than acute coronary heart disease (34–37). These results suggest that the density of calcification plaques may be protective and that high densities of calcification plaques are associated with stability. The mechanism by which this phenomenon occurs is currently unknown (38), and further investigation is needed.

CAC degree is closely associated with age, sex, and other factors, and the extent and prevalence of calcification increase with age (39). CCTA can detect CAC before patient symptoms appear, thus shortening hospital stay and saving costs (40). However, its main disadvantages are the need for iodized contrast media and radiation exposure, and consequently, there may be a potential risk of radiation-related malignant tumors (41).

## What are the Inadequacies and Weak Points of Calcification Detection by Existing CT?

Depending on the vessel site involved, vascular calcification can be classified into medial and intimal calcification (42). As the spatial resolution of normal CT is not ideal, distinguishing between intimal and medial calcification is challenging. Normal CT does not detect microcalcification effectively (8). Additionally, with the widespread use of CT in vascular calcification imaging, radiation exposure is a significant concern. Consequently, reducing radiation exposure while maintaining image quality has become the focus of subsequent technical improvements (43). Currently, four techniques are used to optimize the radiation dose in multi-slice spiral CT (MSCT) arterial imaging: an ECG control tube technique, automatic exposure technique, tube voltage adjustment, and extensive pitch selection technique. It has also been proved that (44) 640-slice CT technology can reduce the radiation dose using wide-area detector technology. However, the expensive CT equipment and high detection cost also limit its wider use (45).

Imaging by CT, high-risk plaques include napkin ring signs, spot calcification, low attenuation plaques, and positive remodeling. The density of the napkin ring sign area is characterized by a high outer and low inner density (46), spot calcification of <3 mm (47), low attenuation feature patches <150 HU (48), and positive remodeling RI>1.1 (47). If two of these are satisfied, it is defined as high-risk plaque (47). High-risk plaques are undesirable characteristics and can significantly increase the risk of cardiovascular disease. Contrastingly, significant calcification can stabilizes the plaque and rarely rupture, reflecting the disease stability (49).

## Current Clinically Available CT Techniques

### MSCT

With the development of medical technology, MSCT has been constantly updated and improved, expanding from its previous 16-slice system to 128-, 256-, 320-, and 640-slice, or more (21). The development of multilayer spiral CT significantly reduces the patient's breath-holding time. At the same time, the improvement in temporal resolution reduces cardiac motion artifacts (50). After long-term clinical application, MSCT is a non-invasive, highly safe, operationally simple technique that can analyze coronary artery calcification and accurately assess the severity of coronary artery stenosis and plaque composition (21). This provides a variety of information for follow-up treatments.

With the gradual improvement in spatial resolution, MSCT allows the complete imaging analysis of minute calcification, and repeated examinations can be performed (21). MSCT is not only fast, simple, and non-invasive with high diagnostic accuracy but also uses many post-processing technologies (51), including volume rendering (VR), maximum intensity projection (MIP), multiplanar reconstruction (MPR), and curved planar reconstruction (CPR). However, these post-processing techniques also have their shortcomings. The role of each post-processing technique is different; for example, VR is beneficial to the observation of the overall arterial anatomy, while CPR is useful in plaque estimation (51). Many post-processing techniques are often used in combination to avoid misdiagnosis or no diagnosis at all.

Over the past decade, MSCT techniques can accurately detect and quantify the degree of arterial calcification. It has been used to monitor the progress of vascular calcification and evaluate and compare the effectiveness of various treatment regimens (45), including assessing the role of vitamin D in CAC (52). Medical staff can integrate CT into the decision-making process and improve work efficiency. Importantly, MSCT has been used to examine vascular calcification, distinguish between patients with different cardiovascular disease incidences and mortality risks, and conduct timely clinical interventions to improve patient disease management and improve patient care.

The dual-source CT (DSCT) is a new technology developed based on 64-slice spiral CT. DSCT with two x-ray tubes and two detectors and the systems can work simultaneously (53). DSCT improves temporal resolution compared to single-source CT, which allows for imaging at higher heart rates. Table 1 compares the main parameters of the first-, second-, and third-generation DSCT (54, 55). With the introduction of third-generation DSCT, its focus is smaller than that of previous generations. Even small anatomy can be displayed with a superior image quality, compared to the previous CT (56). Recently, it was found that the third-generation DSCT can be combined with tin filtering to reduce the radiation dose during calcification image acquisition (57, 58). In another study, Manta et al. (59) used the third-generation DSCT to image calcification in mice, and the scanning time was only 40 s. The total calcium content of detected calcified aortic plaques was as low as 0.71 μg Ca^2+^/mg, proving the feasibility of imaging human-calcified plaques using the CT system. Moreover, the experimental results of Philip et al. showed that the radiation dose during CCTA, using third-generation DSCT, was reduced, and image quality was better than with the second-generation DSCT. They propose that the latest third-generation DSCT CCTA can be performed on patients with a radiation dose of <1 mSv (60).

**Table 1:** Comparison of first-, second-, and third-generation dual-source CT.

**Main parameter**	**First generation**	**Second generation**	**Third generation**
Minimum frame rotation time (s)	0.33	0.28	0.25
Time resolution (ms)	83	75	66
Max scan speed (cm/s)	20.0	45.8	73.7
Maximum number of image layers (layer)	64	128	96
Optional kV value (KV)	80.100.120.140	70.80.100.120.140	70.80.100.120.140.150
Imaging vision (cm)	26	33	50
Ball tube max. power (kW)	2 × 80	2 × 100	2 × 120
Maximum collimation beam width (mm)	19.2	34.8	52.5

### Dual-Energy CT

Dual-energy CT (DECT), also called (61) spectral CT, can image the exact location using two different kVp so that two other datasets are obtained (62). Presently, DECT uses six methods and techniques (63), namely, dual-source DECT, single-source helical DECT, single-source twin-beam DECT, single-source sequential DECT, single-source rapid switching DECT, and dual-layer DECT. Dual-source DECT has a two-source CT system. Each X-ray tube produces a different X-ray energy spectrum. Single-source helical DECT is performed with two spiral scans under different kVp conditions, and single-source twin-beam DECT features the use of split-filter technology. Single-source sequential DECT data are obtained twice with two different kVp, single-source rapid switching DECT features an immediate change in the tube voltage between 80 and 140 kVp, and dual-layer DECT has a unique dual-layer energy-resolving detector (63).

DECT features two different grades of images which can be obtained by one scan and can distinguish between the calcified and non-calcified plaque as well as show the stenosis degree of coronary artery lumen (64). Another advantage of DECT (65) is that it simplifies the workflow. Its data apply to all imaged patients; there is no need to select patients before scanning. DECT can remove artifacts and clearly show the components that produce artifacts such as coronary calcification and metals (66). DECT application technology (67) includes virtual monochromatic imaging (VMI) and virtual non-contrast (VNC) reconstruction. VMI is the synthetic image at a specific single energy level from two spectral x-ray projections, and VNC removes some imaging components (68). Using VMI to reduce the contrast agent dose is considered one of the most promising DECT applications (67). Additionally, VMI has the advantages of improving image contrast (69) and reducing artifacts (70), consequently improving the overall image quality and diagnostic performance. However, VMI has several limitations in reducing artifacts (71); the effect is best at high keV, but this also suppresses the iodine contrast. In addition, the removal of metal artifacts at high keV depends on the properties of the metal itself. VNC can be used to obtain the calcium score of coronary arteries (72). Compared with using calcium score acquisition alone, the radiation dose was reduced, but the displayed calcification was smaller than the actual calcification (73). Therefore, further research is still needed to determine the specific deviation of the VNC from reality (68). In a phantom study, researchers used a plaque similar to human vascular calcification and demonstrated strong agreement between the calcium integration calculated by DECT and the conventional mono-energy CT (74). It takes some time to use these techniques in fast-paced clinical work, which makes it challenging to get the maximum advantages of DECT (75).

DECT uses material decomposition to reduce the calcified components of plaque, thereby improving the visualization of the optical cavity (68). It also enhances plaque visualization to enable the accurate assessment of high-risk plaque features (76). DECT achieves a high-pitch spiral acquisition protocol of 3.0 and higher, shortens the scanning time, and thus reduces the effective radiation dose (77). DECT has a unique acquisition method called high-pitch spiral acquisition. It has a pair of dual-energy detectors. It only uses a quarter of the rotation time to obtain an image, thus providing a higher time resolution (78). According to a study (79), the imaging of ultrasmall superparamagnetic iron oxide by DECT may be helpful to visualize and quantify the accumulation of macrophages in plaque. It is expected that this technique will become a new technique in coronary plaque imaging. Ultrasmall superparamagnetic iron oxide is a negatively charged contrast agent that can stay in the circulatory system for a long time (80).

Logically, the total radiation dose of DECT is twice that of traditional scanning because it needs to obtain data at two different energy levels. However, DECT uses some methods to divide the total radiation dose into high- and low-energy components so that the total dose is no higher than in conventional scanning (81, 82). DECT can subtract calcified plaques from the images, which may improve the assessment of the vascular system, especially with severely calcified plaques (71). Similarly, Domenic et al. (83) evaluated the DECT scanning calcium subtraction algorithm and its influence on the intracavity visualization of patients with severely calcified coronary arteries. They found that compared with the standard linear mixed non-subtraction image, the image with calcium subtraction provided better visibility of the coronary artery lumen and improved the reliability of diagnosis without affecting image quality and contrast noise. However, Michael et al. (84) found that DECT is not effective in evaluating the integrity of blood vessels and the plaque subtraction results are biased. Consequently, additional research is also needed to assess the role of this technique under specific clinical conditions.

In conclusion, further research is still needed in using DECT to identify coronary plaque and evaluate its diagnostic performance and potential clinical value.

## Current Available non-Clinical New CT Techniques

### Micro-CT

Micro-CT has received wide attention as a newly developed imaging method for examining vascular calcification. Microcalcification in the fibrous cap destroys plaque stability by promoting rupture and is not easily detected by the two-dimensional histological method (85). A microcalcification size of only between 5 and 65 μm is sufficient to make the plaque unstable (86). However, ordinary CT cannot effectively detect microcalcification due to spatial resolution limitations. The spatial resolution of micro-CT can reach 1–10 μm (87), which can distinguish and quantify microscopic and macroscopic calcification. Moreover, micro-CT can detect microcalcifications in blood vessels which are normally difficult to find. In animal research, histological methods have been used for calcification analysis, but they have some shortcomings, such as the inability to check for complete vascular calcification. However, using micro-CT surmounts these limitations and calcification can be visualized and quantified three-dimensionally (88). Micro-CT can quantify calcification volume and calcification load, and there was no experimental deviation in the localization and distribution of calcification (88). Calcification load has a strong correlation with the calcification score. The volume represents the spatial size of the calcification plaque and the unit of calcification volume in mm^3^ (89). Although micro-CT is reliable in detecting calcified plaque, it cannot effectively visualize the calcified internal structure, limiting the imaging of small structures such as calcified cell recesses and cell cracks (90). As previously stated, the disadvantages of micro-CT (91) include a long acquisition time, the need for deep anesthesia, poor soft tissue contrast, and high radiation dose, and the radiation doses can reach 760 mGy per scan. However, the CCTA radiation dose is usually around 100–450 mGy (92).

Non-destructive 3D micro-CT has been used in some studies on preclinical vascular calcification. Current CT 3D imaging technology can completely reconstruct calcified arteries and provide accurate quantitative information. 3D micro-CT can detect arterial calcification with an intact vascular structure and accurately quantify calcification by the threshold method (93). 3D micro-CT can be combined with histology, immunohistochemistry, and proteomic methods and can be used as a supplementary means of histological examination. It provides a method of obtaining additional information about calcification volume and load from the same artery segment of the same animal (88). Moreover, the increasing practicality and technological development of the 3D Micro CT also provides a new opportunity to visualize and quantify intimal and medial calcification.

### Carbon Nanotube–Based Micro-CT

Carbon nanotube-based (CNT) micro-CT can accurately generate microsecond transmission pulses and control transmission rays and provide higher temporal and spatial resolution. Compared with traditional micro-CT, the sharpness of the CNT calcification image with micro-CT in mouse models increased (94). This is a helpful tool for evaluating vascular calcification in living mice. However, only a few relevant studies have been conducted, and further research is needed to ensure clinical availability.

### Combining Micro-CT With 18F-NaF Micro-PET/CT

^18^F-NaF micro-PET/CT can distinguish between macrocalcification and microcalcification (95). ^18^F-NaF can bind to the calcified surface of small blood vessels (96). It is an essential method in measuring the calcified surface area and metabolic activity degree of calcified plaque according to the uptake of ^18^F-NaF (97). This is an area where micro-CT does not perform well. Therefore, combining micro-CT with ^18^F-NaF micro-PET/CT may become an excellent method to detect vascular calcification. However, its use may be inhibited by the expensive costs and the lack of standard analysis protocols in its clinical application (98).

### Nano-CT

The spatial resolution of nano-CT can be as high as 400 nm, which exceeds micro-CT and can be imaged in the submicron range (90). With nano-CT imaging, the non-calcified groove of a single plaque cell can be detected, and its histopathological correlation corresponds to chondrocyte-like cells. Currently, the possibility of using nano-CT in the body is remote, as it requires inhibiting human physiological activity. Hence, the technology is likely to be used as an imaging method for *in vitro* analysis (90).

### Synchrotron Radiation CT

The essence of a synchrotron is a circulating particle accelerator, and the X-rays generated by the synchrotron can be used to create 3D images with resolution up to 1 μm (99). Synchrotron radiation CT is an imaging technology belonging to phase-contrast computed tomography (PCCT), combining phase contrast with micron resolution, promoting superior spatial resolution. Differential phase-contrast imaging, a newly developed synchrotron imaging technology, allows the evaluation of large structures and microscopic details of mouse atherosclerotic plaques and is a detailed three-dimensional morphological evaluation. The three-dimensional characteristics of imaging technology also allow detailed evaluation from different angles (100).

PCCT has high spatial resolution and soft-tissue contrast, which can accurately estimate the constituent and shape of plaque, and reliably classify it, and the results are consistent with histopathology (101). Some studies have shown that (102) phase-contrast CT can accurately identify lipid-rich, fibrous, or calcified plaques and has high diagnostic accuracy (sensitivity ≥0.95; specificity ≥0.94). Pfeiffer et al. (103) used phase-contrast CT to examine coronary arteries. The images showed densely calcified plaques and various narrow areas, which were difficult to identify with conventional CT examination (104). However, this technology has not been applied in humans, only *in vitro* or animal research (105).

Future micro-CT studies are needed if its use can be extended to clinical practice. Suppose micro-CT can suitably integrate three-dimensional data of vascular calcification with a manageable and analyzable platform. In that case, it can be more widely applied and is expected to become a conventional method for vascular calcification analysis in the future.

### Ultra-High Resolution CT

The recently developed ultra-high resolution CT (106) allows images with a slice thickness of 0.25 mm, with a higher spatial resolution than traditional CT, and improves the diagnostic accuracy of CCTA in coronary heart disease and the evaluation of coronary stenosis. However, it has the disadvantage of increasing image noise, and the radiation dose is higher than in CT with the latest wide-coverage detector. A recent study shows that (107) material density imaging based on iodine and calcium improves the diagnostic ability of calcified coronary artery disease in patients with a high calcification score. In recent years, subtraction technology has been combined with CCTA. By removing the interference of artifacts caused by calcified plaque and metal stents, subtraction CCTA can improve the accuracy and efficiency of assessing the stenosis of diseased coronary artery segments (108). Recently, pieces of literature have reported that compared with traditional CCTA, subtraction CCTA can significantly improve the imaging quality of coronary artery calcification.

3D virtual intravascular endoscopy is a less invasive tool, which can be used to analyze the morphology of calcified coronary plaque and improve the assessment of coronary stenosis by CCTA (109).

## Conclusion and Prospect

With the development of science and society, imaging inspection equipment, such as CT technology, has been continuously developing since its advent. Different types of CT have their advantages and disadvantages in evaluating vascular calcification (Table 2). Vascular calcification is related to many diseases which seriously affect human health and life. Therefore, early detection and treatment of calcification are of great significance. KDIGO experts noted that any patient with vascular calcification that might affect treatment decisions might require an assessment of vascular calcification (45). Increasingly perfect CT examination makes the diagnostic information captured from images by doctors more accurate and convenient. Also, the evaluation technology of vascular calcification is gradually improving. In the future, it is hoped that CT technology will continue to develop and eventually combine high-definition and low radiation exposure. This will lead to a more extensive application and consequently bring new hope to more patients.

**Table 2:** Comparison of different CT technologies.

**CT type**	**Advantages**	**Disadvantages**	**Macrocalcification**	**Microcalcification**
Dual energy CT	Ability to eliminate artifacts Reduce radiation dose (limited) Improve image quality and contrast Less contrast dose Simplify the workflow	Assess poor vascular integrity Proof of VNC images in calcification less evidence of role	YES	NO
Multi-layer spiral CT	Some post-processing technologies Non-invasive; Simple; fast high diagnostic accuracy	Radiation exposure Increasing odds of associated cancer	YES	NO
Micro-CT	High spatial resolution Microcalcification can be detected	Long acquisition time Need deep anesthesia Poor contrast of the soft tissue High radiation dose Not in clinical	YES	YES

## Author Contributions

ZW and LZ conceived the topic and wrote the first draft. LL, GF, TF, and HJ went through the manuscript and tables. All authors contributed to the article and approved the submitted version.

## Funding

This work was supported as follows the National Natural Science Foundation of China (82070455 and 81770450) the Projects from Social Development of Zhenjiang (SH2019087 and SH2018030).

## Conflict of Interest

The authors declare that the research was conducted in the absence of any commercial or financial relationships that could be construed as a potential conflict of interest.

## Publisher's Note

All claims expressed in this article are solely those of the authors and do not necessarily represent those of their affiliated organizations, or those of the publisher, the editors and the reviewers. Any product that may be evaluated in this article, or claim that may be made by its manufacturer, is not guaranteed or endorsed by the publisher.
